# Patogênese, Avaliação e Tratamento da Disfunção da Microcirculação Coronariana

**DOI:** 10.36660/abc.20230767

**Published:** 2024-08-15

**Authors:** Bing Ji, Xue-Bo Liu

**Affiliations:** 1 Tongji University Tongji Hospital Shanghai China Tongji University – Tongji Hospital, Shanghai – China; 2 Tongji University Department of Cardiology Shanghai China Tongji University – Department of Cardiology, Shanghai – China

**Keywords:** Circulação Coronária, Reserva Fracionada de Fluxo Miocárdico, Terapêutica

## Abstract

A doença cardiovascular é a causa predominante de mortalidade em escala global. A pesquisa indica que as mulheres, em comparação aos homens, apresentam maior probabilidade de apresentar doença arterial coronariana (DAC) não obstrutiva quando têm sintomas de isquemia miocárdica. Além disso, as mulheres tendem a apresentar uma maior carga de sintomas em relação aos homens e, apesar da presença de doença cardíaca isquêmica, são frequentemente tranquilizadas erroneamente devido à ausência de DAC obstrutiva. Nos casos de cardiopatia isquêmica acompanhada de sintomas de isquemia miocárdica, mas sem DAC obstrutiva, é imperativo considerar a disfunção microvascular coronariana como uma potencial causa subjacente. A disfunção microvascular coronariana, caracterizada por reserva de fluxo coronariano prejudicada resultante de anormalidades funcionais e/ou estruturais na microcirculação, está associada a desfechos cardiovasculares adversos. Modificações no estilo de vida e o uso de medicamentos antiateroscleróticos e antianginosos podem oferecer benefícios potenciais, embora sejam necessários mais ensaios clínicos para informar estratégias de tratamento. Esta revisão tem como objetivo explorar a prevalência, mecanismos subjacentes, abordagens diagnósticas e intervenções terapêuticas para disfunção microvascular coronariana.

**Figure:**
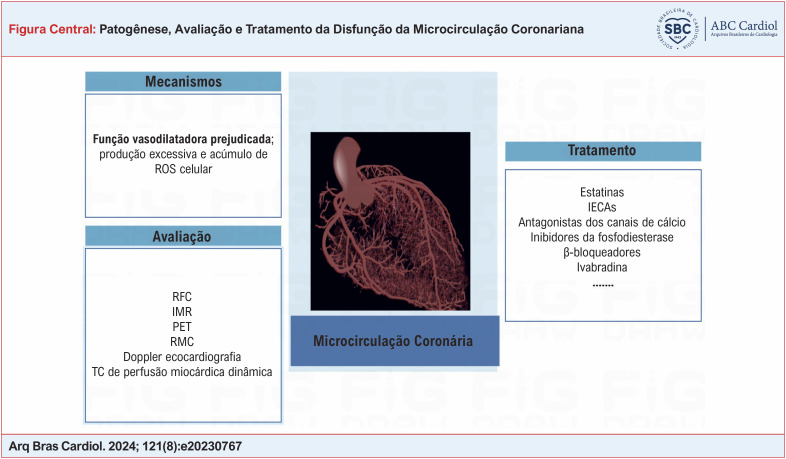
RFC: reserva de fluxo coronariano; IMR: índice de resistência microcirculatória; PET: tomografia por emissão de pósitrons; RMC: ressonância magnética cardíaca; TC: tomografia computadorizada; IECAs: inibidores da enzima de conversão da angiotensina.

## Introdução

Estudos recentes descobriram que uma das principais causas de sintomas clínicos é a doença microvascular coronariana (DMC) e que 39,7% ∼ 62,4% dos pacientes com angina e isquemia não apresentam obstrução vascular epicárdica significativa na angiografia coronariana (estenose visual de <50- 70%). No entanto, há muito se pensa que a doença arterial coronariana (DAC) é uma doença vascular de condução epicárdica para a qual a revascularização mecânica é um tratamento clinicamente eficaz.^1,2^ Acredita-se que a DMC seja um fator que contribui para os sintomas e sinais de isquemia, em oposição à DAC obstrutiva. A DMC é considerada fator contribuinte para os sinais e sintomas de isquemia e não está relacionada à DAC obstrutiva.^1,2^ A DMC parece ser mais prevalente em mulheres do que em homens, sendo as mulheres responsáveis por 70% dos pacientes com DMC.^3^ Entretanto, pacientes do sexo masculino são frequentemente subdiagnosticados em termos de DMC.^4^ Camici e Crea propuseram classificar as DMC em quatro categorias: DMC sem doença miocárdica e DAC obstrutiva, DMC com doença miocárdica, DMC com DAC obstrutiva e DMC congênita.^5^ Este artigo enfoca DMC sem DAC obstrutiva e doença miocárdica, reserva de fluxo coronariano (RFC) prejudicada devido a anormalidades funcionais e/ou estruturais da microcirculação na ausência de hipertrofia ventricular esquerda, cardiomiopatia ou anormalidades valvares.

### Mecanismos fisiológicos da microcirculação coronária

O sistema microcirculatório do coração composto por microveias, microarteríolas e capilares é conhecido como microcirculação coronária do sistema arterial coronariano. DMC refere-se a um grupo de distúrbios que afetam a composição e o funcionamento da microcirculação coronariana, prejudicando o fluxo sanguíneo coronariano e, em última instância, levando à isquemia miocárdica. O sistema arterial coronariano é uma rede contínua que consiste em segmentos vasculares de tamanhos e funções variados. O sistema arterial coronariano consiste em artérias coronárias epicárdicas (> 400 μm), pequenas artérias anteriores (100-400 μm), pequenas artérias intermurais (< 100 μm) e leitos capilares coronários (< 10 μm).^6^ As artérias epicárdicas têm uma função de condução significativa e, em condições normais, proporcionam resistência insignificante ao fluxo sanguíneo coronário, com o diâmetro da artéria epicárdica controlado pelo estresse de cisalhamento e pela função endotelial. Em contraste, as pequenas artérias anteriores e intermurais fornecem a maior parte da resistência ao fluxo sanguíneo coronário e são responsáveis por controlar e distribuir o fluxo sanguíneo para satisfazer as exigências dinâmicas do metabolismo tecidual local através dos capilares coronários. Em condições fisiológicas normais, a perfusão miocárdica está fortemente relacionada com a demanda metabólica, e a resistência vascular coronariana é regulada por alterações no tônus vascular.^7^ Na vasculatura saudável, o fluxo sanguíneo coronário e a perfusão miocárdica são regulados pelo tônus da artéria coronária, com a porção arterial da circulação coronária fornecendo aproximadamente 60% da resistência vascular coronariana, e os capilares e veias fornecendo 25% e 15%, respectivamente, da resistência vascular coronariana. resistência vascular de repouso, que é controlada por controles metabólicos, musculares (dependentes de pressão), endoteliais e neuro-hormonais.^8^ Pequenas arteríolas intermurais menores que 100 μm são reguladas pelo metabolismo tecidual local para garantir que o ambiente extracelular seja ideal para a contração miocárdica.^9^ Pequenas arteríolas intermurais são afetadas principalmente por alterações na pressão de perfusão devido a influências miogênicas, e algumas arteríolas maiores também são afetadas pela pressão de perfusão.^10^ Em contraste, as pequenas arteríolas anteriores não são reguladas pelo ambiente metabólico local e são, portanto, afetadas por outros fatores reguladores. Mais proximalmente, a vasculatura anterior é afetada por mecanismos semelhantes aos da vasculatura epicárdica, principalmente por mecanismos dependentes do endotélio e receptores simpáticos α-1 e α-2 adrenérgicos,^11^ e possivelmente por influências mediadas por receptores β-2 adrenérgicos, que têm também demonstrou desempenhar um papel na função vasomotora epicárdica.^12,13^ A existência de mecanismos autorreguladores bem regulados no sistema coronariano permite a capacidade intrínseca do coração de manter o fluxo sanguíneo apesar das alterações na pressão de perfusão.^14^

### Mecanismos patológicos da doença microvascular coronariana

Vários processos fisiológicos que levam ao aumento da constrição ou à redução da dilatação dos microvasos coronários podem induzir DMC. Uma função vasodilatadora prejudicada pode ser causada por vias não dependentes do endotélio, bem como por disfunção endotelial. Enquanto a último está ligada à diminuição da síntese cíclica de adenosina monofosfato, que resulta em resistência aos nitratos, a primeira está ligada ao diabetes, à obesidade, ao tabagismo e a outros fatores de risco cardiovascular.^15^ Função vasodilatadora prejudicada e/ou vasoconstrição grave resultando em espasmo microvascular são exemplos de alterações funcionais. A disfunção vasodilatadora pode resultar de causas dependentes ou independentes do endotélio.^16,17^ Mediadores vasoconstritores e vasodilatadores, incluindo prostaglandinas, óxido nítrico (NO), fatores hiperpolarizantes derivados do endotélio (EDHFs) e endotelina-1 (ET-1) são produzidos e liberados pelas células endoteliais, que contribuem para a atividade vasomotora. Enquanto os EDHFs são os principais mediadores da vasodilatação microcirculatória coronariana dependente do endotélio, o NO derivado do endotélio causa predominantemente vasodilatação nas artérias coronárias epicárdicas.^18^ O relaxamento reduzido das células endoteliais vasculares, o aumento da liberação de agonistas vasoconstritores (por exemplo, ET-1), o aumento da sensibilidade das células endoteliais vasculares aos estímulos vasoconstritores normais e a atividade autonômica anormal são alguns dos mecanismos dependentes do endotélio que ainda são parcialmente compreendidos.^16,17,19^ Na verdade, a disfunção autonômica, caracterizada como um desequilíbrio entre os sistemas nervoso simpático e parassimpático, tem sido associada ao desenvolvimento de DMC. Este é particularmente o caso em ambientes clínicos onde os mecanismos vasodilatadores já estão comprometidos, como em pacientes com diabetes tipo 2, dislipidemia ou enfarte do miocárdio, bem como nas consequências imediatas destes acontecimentos. O remodelamento hipertrófico para dentro das artérias de resistência coronariana, a diminuição das arteríolas e capilares no lúmen, a fibrose perivascular, a diminuição da densidade dos microvasos e o adelgaçamento capilar são exemplos de alterações microvasculares estruturais que causam DMC.^20^ Essas alterações são mais prevalentes em doenças caracterizadas por hipertrofia ventricular esquerda, como a cardiomiopatia hipertrófica e a cardiopatia hipertensiva.

### Mecanismos moleculares da doença microvascular coronariana

Embora os mecanismos moleculares que levam aos distúrbios da microcirculação coronariana não sejam totalmente compreendidos, o estresse oxidativo e as respostas inflamatórias resultantes da produção e acúmulo excessivo de espécies reativas de oxigênio celular (ROS) são considerados os principais mecanismos patogênicos que levam ao desenvolvimento de distúrbios da microcirculação coronariana.^21^ As células endoteliais desempenham um papel crucial na regulação da vasorreatividade através da liberação de substâncias vasoativas, como NO, que dilata os vasos sanguíneos, e ET-1, que contrai os vasos sanguíneos. As isozimas da nicotinamida adenina dinucleotídeo fosfato oxidase (Nox) e as mitocôndrias são os principais sistemas que regulam a geração de ROS.^22^ A ativação de Nox leva à produção de ROS e desencadeia a fosforilação da proteína adaptadora homóloga de colágeno homólogo Src (p66Shc) e a translocação dentro das mitocôndrias. Em mamíferos, a p66Shc é uma proteína pró-apoptótica que promove ainda mais a produção de ROS, alterando as propriedades biológicas mitocondriais.^21^ Consequentemente, a ativação de p66Shc estimula a atividade Nox, o que leva a um ciclo vicioso de aumento de ROS. Estudos in vitro e vivo demonstraram que o aumento da concentração intracelular de ROS promove a conversão de NO em radicais peroxinitrito e inativa a NO sintase endotelial, mudando sua atividade de uma enzima produtora de NO para uma enzima produtora de ROS, o que leva à vasodilatação mediada por NO prejudicada. e aumenta a atividade vasoconstritora da ET-1 através da ativação da via RhoA/Rho quinase.^23,24^ Além disso, modificações epigenéticas comuns durante o envelhecimento aumentam a produção de ROS, diminuem a expressão de enzimas antioxidantes e promovem a produção de citocinas pró-inflamatórias, ativando a expressão do fator nuclear κ, intensificador da cadeia leve e moléculas de adesão em células B ativadas, sustentando ainda mais o estresse oxidativo.^21^ RhoA/Rho quinase é outra via que está intimamente ligada à síntese de ROS, que é mediada pela regulação da sensibilidade ao cálcio e fosforilação de miofilamentos contráteis, que regula a contratilidade do músculo liso e, portanto, está intimamente associada à hipercontração das células musculares lisas vasculares (CMLV). Assim, acredita-se que a RhoA/Rho quinase seja responsável pelo espasmo que ocorre na vasculatura coronária^24^ e exacerba a inflamação ao induzir fatores pró-inflamatórios nas células do músculo liso vascular e nas células endoteliais.

### Técnicas de diagnóstico

Faltam métodos de triagem para visualizar a microcirculação, o que se reflete principalmente indiretamente na avaliação da função microvascular. A Tabela 1 resume vários métodos para avaliar a função microvascular coronariana (Tabela 1).

**Tabela 1:** Modalidades de avaliação da função microvascular coronariana

Avaliação da Microcirculação Coronária		
Métodos de avaliação invasivos		
	Definição	Desvantagens
RFC	Proporção entre o pico de fluxo de enchimento e o fluxo de repouso	Pouco reprodutível e afetado por estenose epicárdica.
IMR	Produto do tempo médio de condução (Tmn) e da pressão intracoronária distal (Pd) para injeção intracoronária de solução salina por projétil em congestão máxima	Invasivo
**Métodos de avaliação não invasivos**		
	**Vantagens**	**Desvantagens**
PET	O método não invasivo mais eficaz e preciso	Caro e demorado.
RMC	Alta resolução espacial, sem radiação ionizante	O pós-processamento é tecnicamente exigente e demorado
Ecodopplercardiografia da artéria coronária descendente anterior esquerda	Baixo custo, sem radiação ionizante	A precisão é suscetível à proficiência do operador
TC de perfusão miocárdica dinâmica	Maior resolução	Alta dose de radiação

1 RFC: reserva de fluxo coronariano; IMR: índice de resistência microcirculatória; PET: tomografia por emissão de pósitrons; RMC: ressonância magnética cardíaca; TC: tomografia computadorizada.

#### Reserva de fluxo coronário

A RFC é calculada como a razão entre o fluxo sanguíneo coronariano no estado de dilatação máxima e o fluxo sanguíneo coronariano no estado de repouso, e é uma medida da DMC. Uma grande proporção de pacientes apresenta DMC na presença de DAC não obstrutiva difusa. A quantificação da isquemia miocárdica regional e da RFC regional desempenha um papel importante na avaliação da DAC obstrutiva focal. O fluxo sanguíneo coronariano humano pode aumentar de três a quatro vezes durante a isquemia, e medicamentos como adenosina e opioides podem ser usados para induzir congestão máxima na detecção de RFC.^25^ Na ausência de doença vascular epicárdica, a RFC reflete a função microvascular, e é geralmente aceito que existe microcirculação coronária anormal na RFC.

Em repouso, o fluxo sanguíneo coronário depende dos principais fatores que determinam a procura miocárdica de oxigénio, nomeadamente frequência cardíaca, contratilidade e carga ventricular. Entretanto, quando a demanda miocárdica de oxigênio é constante e dentro da faixa de autorregulação, o fluxo sanguíneo coronariano é independente da pressão de perfusão e varia linearmente com a pressão de perfusão durante a fase congestiva máxima de dilatação máxima dos vasos de resistência. Portanto, como a RFC é a razão entre o pico de fluxo de enchimento e o fluxo de repouso, ela é influenciada por fatores do fluxo sanguíneo coronário em repouso que afetam a repetibilidade da relação.^26^ Além disso, a doença vascular epicárdica pode ter um impacto significativo na proporção, tornando-se um método não quantificável de quantificação da doença microvascular para a maioria dos pacientes cardíacos.

#### Índice de resistência microcirculatória

O índice de resistência microcirculatória (IMR) é uma medida de resistência microvascular mínima e função microvascular, semelhante à RFC derivado da termodiluição, que utiliza o fio guia sensor de temperatura e pressão (TPSG) para medir simultaneamente o tempo médio de condução (Tmn) de solução salina injetado nas artérias coronárias por um projétil durante a congestão máxima e a pressão intracoronária distal (Pd), sendo a IMR o produto desses dois parâmetros, Tmn e Pd.

Usando um modelo suíno, Fearon et al. compararam a verdadeira resistência microvascular (RMV) na presença e ausência de disfunção microvascular, investigando a disfunção microvascular gerada artificialmente através do uso de microesferas injetadas nas artérias coronárias, sendo a verdadeira resistência microvascular definida como a pressão distal à LAD dividida pela coronária absoluta fluxo sanguíneo derivado usando uma sonda de fluxo ultrassônica, e os pesquisadores descobriram que uma correlação razoável entre IMR e RMV estava razoavelmente correlacionada (r = 0,54 p < 0,0001), com valores de IMR aumentando com a deterioração da função microvascular, independente da estenose epicárdica.^27^

Alguns investigadores descobriram que, na presença de estenose grave, ignorar o aumento do fluxo colateral pode levar a uma superestimação da resistência microvascular. Portanto, na presença de estenose epicárdica, a fórmula IMR é modificada da seguinte forma: Pa*Tmn (Pd - Pw / Pa - Pw), onde Pa é a alta pressão aórtica, Pd é a alta pressão distal à estenose, e Pw é a pressão de cunha coronária (definida como a pressão média da artéria coronária distal no vaso alvo durante a oclusão do balão).^28^ Como mencionado anteriormente, a correção da pressão de cunha coronária é necessária para pacientes com estenose hemodinâmica significativa. Em geral, quanto maior a TMI, pior é a função microcirculatória. Valores inferiores a 20 são considerados normais, enquanto valores superiores a 30 são geralmente considerados anormais, mas há uma sobreposição considerável entre os dois, e a disfunção microcirculatória coronária é agora considerada principalmente presente com uma IMR ≥25.^29^

caIMR (índice de resistência microcirculatória derivado da angiografia coronária) é um novo método de avaliação não invasivo baseado em imagens de angiografia coronária, que não requer fio-guia de pressão e estado congestivo máximo induzido por drogas para cálculo de IMR, e foi demonstrado que caIMR é semelhante à IMR em isquemia sem doença coronariana obstrutiva(INOCA)pacientes. Uma boa correlação entre caIMR e IMR foi demonstrada em pacientes INOCA.^30^ Outro estudo, o estudo FLASH IMR, relatou novamente boa acurácia diagnóstica do caIMR,^31^ mas o número de casos incluídos nele ainda era pequeno. Uma metanálise recente reuniu todos os métodos acima de IMR derivados angiograficamente e descobriu que todos eles tinham alta precisão diagnóstica e eram preditores independentes de futuros eventos cardíacos adversos.^32^ No entanto, os tamanhos das amostras dos ensaios incluídos na metanálise foram todos pequenos, variando de 25 a 262 pacientes e, portanto, são aguardados estudos clínicos maiores para a precisão da determinação da IMR apenas pela angiografia.

#### Tomografia por emissão de pósitrons

A tomografia por emissão de pósitrons (PET) é o método não invasivo mais eficaz e preciso para avaliar quantitativamente a função vasomotora coronariana. Com os avanços da tecnologia, essas medidas foram incorporadas aos testes de carga de perfusão miocárdica PET de rotina.^33^ Cada estudo é realizado após a injeção de um radiotraçador de fluxo sanguíneo (82Rb e 13N), e o pós-processamento das imagens de repouso e de carga permite a quantificação do fluxo sanguíneo miocárdico regional e global (em ml/min/g de miocárdio) e o cálculo da RFC (a relação entre carga e fluxo sanguíneo miocárdico em repouso). Dados recentes demonstraram que a DMC quantificada como RFC reduzida é comum em pacientes com DAC conhecida ou suspeita,^34^ aumenta a gravidade da isquemia miocárdica induzível (além dos efeitos da obstrução da artéria coronária epicárdica) e da lesão miocárdica subclínica,^35^ e identifica pacientes com alto risco de MACE, incluindo morte cardíaca.^36,37^

#### Ressonância magnética cardíaca

A ressonância magnética cardíaca (RMC) pode ser usada para quantificar a perfusão miocárdica de maneira semelhante à PET, mas a técnica de pós-processamento é tecnicamente exigente e demorada. Tal como acontece com a PET, o protocolo de imagem inclui repouso e carga, sendo as imagens de carga realizadas após a injeção de contraste de gadolínio. Após o pós-processamento das imagens de repouso e de carga, a perfusão miocárdica regional e global pode ser quantificada usando modelos semiquantitativos (índice de reserva de perfusão miocárdica) ou totalmente quantitativos (RFC). Um método de RMC sem carga de gadolínio utilizando mapeamento T1 também foi proposto recentemente para o diagnóstico de isquemia miocárdica com ou sem DAC obstrutiva.^38^ A RMC tem a vantagem de alta resolução espacial para caracterização transmural do fluxo sanguíneo miocárdico e ausência de radiação ionizante, avaliação da DMC por uma redução no índice de reserva de perfusão miocárdica, uma avaliação abrangente da estrutura e função cardiovascular, e tem se mostrado preditiva de prognóstico.^39^ No entanto, os dados permanecem limitados.

#### Ecocardiografia Doppler coronária da descendente anterior esquerda

Ecocardiografia Doppler coronária da LAD pode ser usada para quantificar a velocidade do fluxo sanguíneo coronariano em repouso e durante a carga vasodilatadora. A velocidade do fluxo sanguíneo coronário é medida por Doppler de onda pulsada e avaliada como velocidade máxima do fluxo sanguíneo diastólico em repouso e enchimento máximo. A reserva de velocidade do fluxo coronário é calculada como a razão entre a velocidade do fluxo coronário na congestão máxima e a velocidade do fluxo coronário em repouso. As vantagens desta técnica são que ela é de baixo custo, livre de radiação ionizante e amplamente disponível, mas a precisão é suscetível à proficiência do operador e requer visualização ecocardiográfica das artérias coronárias proximais, o que pode ser um desafio significativo em adultos obesos. Há evidências crescentes de que um índice de reserva de velocidade de fluxo coronariano reduzido pode ajudar a identificar e estratificar o risco de DMC.^40,41^

#### Tomografia computadorizada de perfusão miocárdica dinâmica

A tomografia computadorizada (TC) de perfusão miocárdica dinâmica pode ser usada para estimar o fluxo sanguíneo miocárdico de maneira semelhante à imagem de perfusão RMC. A TC dinâmica é realizada após a injeção de um agente de contraste iodado e utiliza aquisição dinâmica de imagens para obter uma estimativa do fluxo sanguíneo miocárdico da mesma forma que a RMC.^33^ As principais vantagens desta técnica são a alta resolução espacial da TC e a capacidade de fornecer avaliação anatômica e funcional precisa do miocárdio e das artérias coronárias em um único exame. No entanto, essas vantagens acarretam o custo de uma dose de radiação mais elevada para o paciente.

### Tratamento

#### Estatinas

Em pequenos ensaios, estes tratamentos melhoraram os sintomas de angina, a perfusão miocárdica, a função endotelial coronária e a função microvascular. As estatinas reduzem núcleos ricos em lipídios em placas, inflamação, macrófagos e formação de células espumosas, promovem espessamento da capa fibrosa e reduzem a reatividade plaquetária.^42-44^ As estatinas são eficazes na redução dos níveis de LDL, reduzindo assim o risco cardiovascular. Além disso, as estatinas podem ter múltiplos efeitos, incluindo a redução da inflamação vascular e a melhoria da função endotelial. Os tratamentos atuais de CMD focam no controle de fatores de risco e no alívio de sintomas, e há uma falta de recomendações baseadas em evidências de alto nível. A Tabela 2 resume vários tratamentos para CMD.

**Tabela 2:** Tratamento da Microcirculação Coronária disfunção

Tratamento da Disfunção da Microcirculação Coronariana	
Medicamentos	Mecanismo
Estatinas	reduzindo a inflamação vascular e melhorando a função endotelial.
IECAs	mediar indiretamente os efeitos na função microvascular coronariana, reduzindo a pressão arterial
Antagonistas dos canais de cálcio	melhorar a capacidade de vasodilatação microcirculatória coronária e reduzir a pós-carga cardíaca
Inibidor da fosfodiesterase	artérias coronárias dilatadas e melhora da vasodilatação dependente do endotélio
β bloqueadores	CFVR regional aumentou significativamente em segmentos estenóticos
Ivabradina	melhorar a tolerância ao exercício, prolongar o tempo isquêmico durante o exercício

IECAs: inibidores da enzima conversora de angiotensina.

Em um estudo duplo-cego, randomizado e controlado por placebo realizado por Kayikcioglu et al.,^45^ 40 pacientes com angina microvascular foram randomizados para tomar pravastatina (40 mg/dia) ou placebo. Após 3 meses de tratamento, a dilatação mediada pelo fluxo da artéria braquial melhorou significativamente no grupo da pravastatina. Da mesma forma, a duração do exercício e a inibição de 1 mm-ST foram significativamente prolongadas após o tratamento com estatinas em comparação com o placebo. Em um estudo semelhante, Fabian et al. avaliaram 40 pacientes com angina microvascular que apresentavam hipercolesterolemia leve e foram randomizados para receber placebo (20) ou sinvastatina 20 mg/dia (20).^46^ No final do estudo, a dilatação mediada pelo fluxo da artéria braquial aumentou significativamente no grupo de tratamento, e o tempo para depressão do segmento ST> 1 mm no teste de esforço foi significativamente prolongado. Mais recentemente, Zhang et al. avaliaram o efeito da terapia combinada com estatinas e bloqueadores dos canais de cálcio versus monoterapia em pacientes com angina microvascular.^47^ Sessenta e oito pacientes foram randomizados em três grupos: fluvastatina (40 mg/dia, 23), diltiazem (90 mg/dia, 22) e combinação fluvastatina (40 mg/dia) e diltiazem (90 mg/dia, 23). A RFC melhorou nos três grupos após 90 dias. Além disso, o tempo para depressão do segmento ST de 1 mm foi significativamente maior em todos os grupos. A melhoria na reserva de fluxo coronário e o prolongamento do tempo até a depressão do segmento ST de 1 mm foram mais pronunciados no grupo de combinação do que nos pacientes que receberam monoterapia. Com base nestes estudos, recomendamos o uso de estatinas na maioria dos pacientes com doença microvascular, a menos que existam efeitos secundários graves ou contraindicações.

#### Inibidores da enzima conversora de angiotensina

Em um estudo sobre medicamentos ativos para o SRAA (incluindo IECA), com pacientes com DMC usando medidas Doppler de RFC nas artérias intracoronárias, um ensaio randomizado controlado de terapia com IECA para DMC confirmou o benefício do enalapril. Em indivíduos diabéticos, o IECA melhorou a RFC, e em pacientes hipertensos, o IECA demonstrou melhorar a função microvascular coronariana pela PET,^48^ mas nenhum efeito foi encontrado no estudo de Kawata et al.^49^ Estudos não randomizados utilizando diferentes métodos para avaliar a função microvascular coronariana em pequenas amostras de pacientes confirmaram ainda que os IECA melhoram a função microvascular coronariana; é incerto se os IECA medeiam indiretamente os efeitos na função microvascular coronária através da redução da pressão arterial, mas este pode não ser um mecanismo único de benefício, dado o papel fundamental da angiotensina II na função vascular.^50^ Outras evidências sugerem que os IECA estão associados a melhorias nos marcadores da função endotelial e em vários biomarcadores circulantes. Em outro estudo randomizado, duplo-cego, controlado por placebo em pacientes do sexo feminino com isquemia com DAC não obstrutiva (INOCA), a adição de um antagonista seletivo do receptor de aldosterona (eplerenona) a um IECA não reduziu os sintomas de angina nem melhorou a RFC.^51^

#### Antagonistas dos canais de cálcio

Os antagonistas dos canais de cálcio são amplamente utilizados no tratamento da angina vasospástica (AVS). A benidipina, uma dihidropiridina de ação prolongada, produziu efeitos prognósticos benéficos em pacientes com AVS.^52^ Os antagonistas do cálcio melhoram a capacidade de vasodilatação da microcirculação coronariana e reduzem a pós-carga cardíaca e, portanto, são frequentemente usados em pacientes com DMC. Além disso, a nifedipina de ação prolongada pode exercer efeitos cardioprotetores ao inibir a inflamação vascular e melhorar a função endotelial em pacientes com angina ou isquemia sem doença coronariana obstrutiva (ANOCA/INOCA).^53^ Contudo, o uso de amlodipina em pacientes INOCA não melhorou significativamente a angina de peito e, em outro estudo, o verapamil não conseguiu reduzir as alterações do segmento ST no ECG devido à isquemia.^54^ Assim, os bloqueadores dos canais de cálcio do tipo L de ação prolongada parecem ser mais favoráveis à microcirculação coronária do que os bloqueadores dos canais de cálcio do tipo L de ação curta.

#### O inibidor da fosfodiesterase

O inibidor da fosfodiesterase (PDE) tipo 3, cilostazol, é usado na claudicação intermitente e na prevenção de pós-AVC, reestenose de stent coronário e intervenção coronária percutânea. O receptor PDE-3 é subdividido em PDE-3A, que é encontrado em plaquetas, células musculares lisas vasculares e cardiomiócitos, e PDE-3B, que é encontrado em adipócitos, hepatócitos, células β pancreáticas e macrófagos. Este último é encontrado em adipócitos, hepatócitos, células β pancreáticas e macrófagos. A inibição da PDE aumenta o AMP cíclico intracelular, que tem efeitos antiplaquetários, antiinflamatórios e vasodilatadores. O cilostazol reduz o ânion superóxido e melhora a produção local do fator de crescimento dos hepatócitos, bem como a motilidade e morfogênese das células epiteliais e endoteliais.^55^ A adição de cilostazol pareceu ser eficaz em um estudo multicêntrico prospectivo de pacientes com AVS refratária a nitratos induzidos por antagonistas de cálcio e espontâneos ou ergonovina.^56^ Num ensaio multicêntrico, randomizado, duplo-cego e controlado por placebo de amlodipina em pacientes com AVS refratária ao tratamento, o cilostazol reduziu a frequência e a intensidade dos episódios anginosos sem efeitos adversos graves.^57^ Num modelo canino, um inibidor do tipo PDE -5 (sildenafil) melhorou a perfusão do miocárdio hipoperfundido durante o exercício.^58^ Em pacientes com DAC, uma dose única de sildenafil (sem comparação com placebo) dilatou as artérias coronárias e melhorou a vasodilatação dependente do endotélio.^59^

#### β bloqueadores

Embora os primeiros estudos utilizando propranolol e vinblastina não tenham relatado efeitos benéficos,^60,61^ estudos utilizando atenolol relataram resultados favoráveis em termos de sintomas e parâmetros do teste ergométrico.^62^ O atenolol é frequentemente prescrito em doses de até 100 mg por dia. Mais recentemente, relatórios avaliando os efeitos do nebivolol nesses pacientes demonstraram benefícios semelhantes em doses de até 5 mg por dia.^63^ Os betabloqueadores são particularmente eficazes em pacientes com frequência cardíaca em repouso elevada ou tônus simpático aumentado^64^ e devem ser evitados em pacientes com distúrbios vasoespásticos comórbidos.^65^ Assim, dependendo da apresentação clínica e das comorbidades, os betabloqueadores podem muitas vezes ser um tratamento eficaz de DMC e são utilizados como agentes de primeira linha, dependendo da apresentação clínica e das comorbidades.

Um estudo encontrou melhora significativa na função microvascular coronariana após tratamento com carvedilol comparado ao tratamento com metoprolol em pacientes com hipertensão.^66^ Koepfli et al.^66^ não encontraram efeito do carvedilol ou metoprolol em segmentos estenóticos e remotos de artérias coronárias em pacientes com DAC e angina de peito estável, mas uma análise conjunta mostrou que a RVFC regional aumentou significativamente em segmentos estenóticos.^67^ No entanto, se este efeito foi devido à regressão da aterosclerose ou à melhoria da função microvascular coronária permanece desconhecido.

#### Ivabradina

A ivabradina não causa vasoconstrição ou efeitos inotrópicos negativos em comparação com os betabloqueadores. Os efeitos benéficos da ivabradina na doença cardíaca isquêmica (DIC) são mediados por efeitos indiretos, que melhoram a tolerância ao exercício, prolongam o tempo isquêmico durante o exercício e reduzem a gravidade e melhoram a tolerância ao exercício, e prolongam o tempo isquêmico durante o exercício, frequência de angina em pacientes com angina estável. A ivabradina melhorou os sintomas em pacientes com angina de peito microvascular primária, mas a função microvascular coronariana não foi alterada, sugerindo que a melhora dos sintomas pode ser atribuída a um efeito de redução da frequência cardíaca. Outros estudos descobriram que a ivabradina estabiliza a RFC em pacientes com DAC.^68^ Portanto, os bloqueadores de canais podem ter um papel em pacientes com DMC, mas são necessários mais estudos.

## Conclusões

DMC é uma classe de doenças causadas por distúrbios microvasculares coronários estruturais e/ou funcionais com patogênese e procedimentos diagnósticos específicos. A avaliação da função microcirculatória coronariana deve ser o foco da terapia direcionada em pacientes nos quais foram excluídos estenose grave ou espasmo das artérias coronárias epicárdicas, mas que continuam a apresentar episódios de angina. Atualmente, o tratamento da DMC consiste principalmente no controle dos fatores de risco ou no tratamento da doença primária, e a farmacoterapia limitada continua a ser confirmada por novos ensaios clínicos.
