# Supplementary material for: Patogênese, Avaliação e Tratamento da Disfunção da Microcirculação Coronariana
Source: Arq Bras Cardiol. 2024 Aug 15;121(8):e20230767. [Article in Portuguese] doi: 10.36660/abc.20230767 (PMC11495817; doi:10.36660/abc.20230767)
Supplement: Translation

# Translation: Pathogenesis, Assessment, and Treatment of Coronary Microcirculation Dysfunction

**DOI:** 10.36660/abc.20230767i

**Authors:** Bing Ji, Xue-Bo Liu

**Affiliations:** 1 Shanghai China Tongji University – Tongji Hospital, Shanghai – China; 2 Shanghai China Tongji University – Department of Cardiology, Shanghai – China

**Keywords:** Coronary Circulation, Myocardial Fractional Flow Reserve, Therapeutics

## Abstract

Cardiovascular disease is the predominant cause of mortality on a global scale. Research indicates that women exhibit a greater likelihood of presenting with non-obstructive coronary artery disease (CAD) when experiencing symptoms of myocardial ischemia in comparison to men. Additionally, women tend to experience a higher burden of symptoms relative to men, and despite the presence of ischemic heart disease, they are frequently reassured erroneously due to the absence of obstructive CAD. In cases of ischemic heart disease accompanied by symptoms of myocardial ischemia but lacking obstructive CAD, it is imperative to consider coronary microvascular dysfunction as a potential underlying cause. Coronary microvascular dysfunction, characterized by impaired coronary flow reserve resulting from functional and/or structural abnormalities in the microcirculation, is linked to adverse cardiovascular outcomes. Lifestyle modifications and the use of anti-atherosclerotic and anti-anginal medications may offer potential benefits, although further clinical trials are necessary to inform treatment strategies. This review aims to explore the prevalence, underlying mechanisms, diagnostic approaches, and therapeutic interventions for coronary microvascular dysfunction.

**Figure:** CFR: coronary flow reserve; IMR: index of microcirculatory resistance; PET: positron emission tomography; CMR: cardiac magnetic resonance; CT: computed Tomography; ACEIs: angiotensin-converting enzyme inhibitors.

## Introduction

Recent studies have found that one of the main causes of clinical symptoms is Coronary Microvascular Disease (CMD) and that 39.7% ∼ 62.4% of patients with angina and ischemia do not have significant epicardial vascular obstruction on coronary angiography (visual stenosis of <50-70%). However, Coronary artery disease has long been thought to be an epicardial conduction vascular disease for which mechanical revascularization is a clinically effective treatment.^1,2^ CMD is thought to be a contributing factor to the symptoms and signs of ischemia, as opposed to Obstructive Coronary Artery Disease. CMD is considered a contributing factor to the signs and symptoms of ischemia and is not related to Obstructive Coronary Artery Disease.^1,2^ CMD appears to be more prevalent in women than in men, with women accounting for 70% of CMD patients.^3^ However, male patients are often underdiagnosed in CMD.^4^ Camici and Crea proposed to classify CMD into four categories: CMD without myocardial disease and obstructive CAD, CMD with myocardial disease, CMD with obstructive CAD, and congenital CMD^5^. This article focuses on CMD without obstructive CAD and myocardial disease, impaired coronary flow reserve (CFR) due to functional and/or structural abnormalities of the microcirculation in the absence of left ventricular hypertrophy, cardiomyopathy, or valvular abnormalities.

### Physiological mechanisms of coronary microcirculation

The microcirculatory system of the heart made up of microveins, microarterioles, and capillaries is known as the coronary microcirculation of the coronary artery system. CMD refers to a group of disorders that impact the composition and operation of the coronary microcirculation, hence impairing coronary blood flow and ultimately leading to myocardial ischemia. The coronary arterial system is a continuous network consisting of vascular segments of varying sizes and functions. The coronary artery system consists of epicardial coronary arteries (> 400 μm), anterior small arteries (100-400 μm), intermural small arteries (< 100 μm), and coronary capillary beds (< 10 μm).^6^ Epicardial arteries have a significant conduction function and, in normal conditions, provide negligible resistance to coronary blood flow, with epicardial artery diameter controlled by shear stress and endothelial function. In contrast, the anterior and intermural small arteries provide the majority of the resistance to coronary blood flow and are in charge of controlling and distributing blood flow to fulfill the dynamic demands of local tissue metabolism via the coronary capillaries. Under normal physiological conditions, myocardial perfusion is strongly related to metabolic demand, and coronary vascular resistance is regulated by changes in vascular tone.^7^ In healthy vasculature, coronary blood flow and myocardial perfusion are regulated by coronary artery tone, with the arterial portion of the coronary circulation providing approximately 60% of coronary vascular resistance, and the capillaries and veins providing 25% and 15%, respectively, of the resting vascular resistance, which is controlled by metabolic, muscular (pressure-dependent), endothelial, and neurohormonal controls.^8^ Small intermural arterioles smaller than 100 μm are regulated by local tissue metabolism to ensure that the extracellular environment is optimal for myocardial contraction.^9^ Small intermural arterioles are mainly affected by changes in perfusion pressure due to myogenic influences, and some larger arterioles are also affected by perfusion pressure.^10^ In contrast, small anterior arterioles are not regulated by the local metabolic environment and are therefore affected by other regulatory factors. More proximally, the anterior vasculature is affected by mechanisms similar to those of the epicardial vasculature, mainly by endothelium-dependent mechanisms and sympathetic α-1 and α-2 adrenergic receptors,^11^ and possibly by β-2 adrenergic receptor-mediated influences, which have also been shown to play a role in epicardial vasomotor function.^12,13^ The existence of well-regulated autoregulatory mechanisms in the coronary system allows the heart's intrinsic ability to maintain blood flow despite changes in perfusion pressure.^14^

### Pathological mechanisms of CMD

Several physiological processes that lead to either enhanced constriction or reduced coronary microvessel dilatation can induce CMD. An impaired vasodilatory function may be caused by non-endothelium-dependent pathways as well as endothelial dysfunction. While the latter is linked to decreased cyclic adenosine monophosphate synthesis, which results in nitrate resistance, the former is linked to diabetes, obesity, smoking, and other cardiovascular risk factors.^15^ Impaired vasodilatory function and/or severe vasoconstriction resulting in microvascular spasm are examples of functional alterations. Vasodilatory dysfunction may result from endothelium-dependent or -independent causes.^16,17^ Vasoconstrictor and vasodilator mediators, including prostaglandins, nitric oxide (NO), endothelium-derived hyperpolarizing factors (EDHFs), and endothelin-1 (ET-1) are produced and released by endothelial cells, which contribute to vasomotor activity. Whereas EDHFs are the primary mediators of endothelium-dependent coronary microcirculatory vasodilation, endothelium-derived NO predominantly causes vasodilation in epicardial coronary arteries.^18^ Reduced relaxation of vascular endothelial cells, increased release of vasoconstrictor agonists (e.g., ET-1), increased sensitivity of vascular endothelial cells to normal vasoconstrictor stimuli, and abnormal autonomic activity are some of the endothelium-dependent mechanisms that are still partially understood.^16,17,19^ In fact, autonomic dysfunction, which is characterized as an imbalance between the sympathetic and parasympathetic nervous systems, has been linked to the development of CMD. This is particularly the case in clinical settings where vasodilatory mechanisms are already impaired, such as in patients with type 2 diabetes, dyslipidemia, or myocardial infarction, as well as in the immediate aftermath of these events. Hypertrophic inward remodeling of coronary resistance arteries, a decrease in arterioles and capillaries in the lumen, perivascular fibrosis, a decrease in microvessel density, and capillary thinning are examples of the structural microvascular alterations that cause CMD.^20^ These changes are more prevalent in diseases characterized by left ventricular hypertrophy, such as hypertrophic cardiomyopathy and hypertensive heart disease.

### Molecular mechanisms of CMD

Although the molecular mechanisms leading to coronary microcirculatory disorders are not fully understood, oxidative stress and inflammatory responses resulting from excessive production and accumulation of cellular reactive oxygen species (ROS) are considered to be the key pathogenic mechanisms leading to the development of coronary microcirculatory disorders.^21^ Endothelial cells play a crucial role in regulating vasoreactivity through the release of vasoactive substances such as NO, which dilates blood vessels, and ET-1, which constricts blood vessels. Nicotinamide adenine dinucleotide phosphate oxidase (Nox) isozymes and mitochondria are the main systems regulating ROS generation.^22^ Activation of Nox leads to ROS production and triggers phosphorylation of Src homologous-collagen homolog adaptor protein (p66Shc) and translocation within mitochondria. In mammals, p66Shc is a pro-apoptotic protein that further promotes ROS production by altering mitochondrial biological properties.^21^ Accordingly, activation of p66Shc stimulates Nox activity, which leads to a vicious cycle of increased ROS. In vitro and vivo studies have shown that increased intracellular ROS concentration promotes the conversion of NO in peroxynitrite radicals and inactivates endothelial NO synthase, changing its activity from a NO-producing enzyme to a ROS-producing enzyme, which leads to impaired NO-mediated vasodilatation and enhances the vasoconstrictor activity of ET-1 through activation of the RhoA/Rho kinase pathway.^23,24^ In addition, common epigenetic modifications during aging increase ROS production, decrease the expression of antioxidant enzymes and promote pro-inflammatory cytokine production by activating the expression of nuclear factor κ light chain enhancer and adhesion molecules in activated B-cells, further sustaining oxidative stress.^21^ RhoA/Rho kinase is another pathway that is closely linked to ROS synthesis, which is mediated through the regulation of calcium sensitivity and phosphorylation of contractile myofilaments, which regulates smooth muscle contractility and thus is closely associated with vascular smooth muscle cell (VSMC) hypercontraction. Thus, RhoA/Rho kinase is thought to be responsible for the spasm that occurs in the coronary vasculature^24^ and exacerbates inflammation by inducing pro-inflammatory factors in vascular smooth muscle cells and endothelial cells.

### Diagnostic techniques

There is a lack of screening methods to visualize the microcirculation, which is mainly reflected indirectly by assessing microvascular function. Table 1 summarizes several methods for assessing coronary microvascular function (Table 1).

**Table 1:** Modalities to assess coronary microvascular function

Assessment of Coronary Microcirculation		
Invasive assessment methods		
	Definition	Disadvantages
CFR	Ratio of peak filling flow to resting flow	Poorly reproducible and affected by epicardial stenosis.
IMR	Product of mean conduction time (Tmn) and distal intracoronary pressure (Pd) for intracoronary projectile saline injection at maximal congestion	Invasive
**Non-invasive assessment methods**		
	**Advantages**	**Disadvantages**
PET	The most effective and accurate non-invasive method	Expensive and time-consuming.
CMR	High spatial resolution, no ionizing radiation	Post-processing is technically demanding and time-consuming
Doppler echocardiography ofthe left anterior descending coronary artery	Low cost, no ionizing radiation	Accuracy is susceptible to operator proficiency
Dynamic Myocardial Perfusion CT	Higher resolution	High radiation dose

CFR: coronary flow reserve; IMR: index of microcirculatory resistance; PET: positron emission tomography; CMR: cardiac magnetic resonance; CT: computed tomography.

#### Coronary flow reserve

Coronary flow reserve (CFR) is calculated as the ratio of coronary blood flow in the maximally dilated state to coronary blood flow in the resting state, and it is a measure of CMD. A large proportion of patients present with CMD in the presence of diffuse nonobstructive CAD. Quantification of regional myocardial ischemia and regional CFR plays an important role in the assessment of focal obstructive CAD. Human coronary blood flow can increase three- to four-fold during ischemia, and drugs such as adenosine and opioids can be used to induce maximal congestion when detecting CFR.^25^ In the absence of epicardial vascular disease, CFR reflects microvascular function, and it is generally accepted that abnormal coronary microcirculation exists with a CFR.

At rest, coronary blood flow depends on the main factors determining myocardial oxygen demand, namely heart rate, contractility, and ventricular load. However, when myocardial oxygen demand is constant and within the autoregulation range, coronary blood flow is independent of perfusion pressure and varies linearly with perfusion pressure during the maximal congestive phase of maximal dilatation of resistance vessels. Therefore, since CFR is the ratio of peak filling flow to resting flow, it is influenced by factors of resting coronary blood flow that affect the repeatability of the ratio.^26^ In addition, epicardial vascular disease can have a significant impact on the ratio, making it an unquantifiable method of quantifying microvascular disease for most cardiac patients.

#### Index of microcirculatory resistance

The index of microcirculatory resistance (IMR) is a measure of minimal microvascular resistance and microvascular function, similar to the CFR derived from thermodilution, which utilizes the temperature and pressure sensing guidewire(TPSG) to simultaneously measure the mean conduction time (Tmn) of saline injected into the coronary arteries by a projectile during maximal congestion and the distal intracoronary pressure (Pd), with the IMR being the product of these two parameters, Tmn and Pd. Using a porcine model, Fearon et al. compared true microvascular resistance (TMR) in the presence and absence of microvascular dysfunction, investigating artificially generated microvascular dysfunction through the use of microspheres injected into the coronary arteries, with true microvascular resistance being defined as the pressure distal to the LAD divided by the absolute coronary blood flow derived using an ultrasonic flow probe, and the researchers found a reasonable correlation between IMR and TMR were reasonably correlated (r = 0.54 p < 0.0001), with values of IMR increasing with deterioration of microvascular function, independent of epicardial stenosis.^27^

Some investigators have found that in the presence of severe stenosis, ignoring the increase in collateral flow may lead to an overestimation of microvascular resistance. Therefore, in the presence of epicardial stenosis, the IMR formula is modified as follows: Pa*Tmn (Pd - Pw / Pa - Pw), where Pa is the aortic high pressure, Pd is the high pressure distal to the stenosis, and Pw is the coronary wedge pressure (defined as the mean distal coronary artery pressure in the target vessel during balloon occlusion).^28^ As mentioned earlier, correction for coronary wedge pressure is necessary for patients with significant hemodynamic stenosis. In general, the higher the IMR, the worse the microcirculatory function. Values less than 20 are considered normal, whereas values greater than 30 are generally considered abnormal, but there is considerable overlap between the two, and coronary microcirculatory dysfunction is now mostly considered to be present with an IMR ≥25.^29^

caIMR (coronary angiography-derived index of microcirculatory resistance) is a novel noninvasive evaluation method based on coronary angiography images, which does not require a pressure guidewire and drug-induced maximal congestive state for calculation of IMR, and it has been demonstrated that caIMR is similar to IMR in ischemia with no obstructive coronary disease (INOCA) patients. A good correlation between caIMR and IMR has been demonstrated in INOCA patients.^30^ Another study, the FLASH IMR study, again reported good diagnostic accuracy of caIMR,^31^ but the number of cases included in it was still small. A recent meta-analysis pooled all the above methods of angiographically derived IMR and found that all of them had high diagnostic accuracy and were independent predictors of future adverse cardiac events.^32^ However, the sample sizes of the trials included in the meta-analysis were all small, ranging from 25 ∼ 262 patients, and thus larger clinical studies are awaited for the accuracy of IMR determination by angiography alone.

#### Positron emission tomography

Positron emission tomography (PET) is the most effective and accurate non-invasive method for quantitatively assessing coronary vasomotor function. With advances in technology, these measurements have been incorporated into routine PET myocardial perfusion loading tests.^33^ Each study is performed after injection of a blood flow radiotracer (82Rb and 13N), and post-processing of resting and loading images allows quantification of regional and overall myocardial blood flow (in ml/min/g of myocardium) and calculation of CFR (the ratio of load to resting myocardial blood flow). Recent data have demonstrated that CMD quantified as reduced CFR is common in patients with known or suspected CAD,^34^ increases the severity of inducible myocardial ischemia (beyond the effects of epicardial coronary artery obstruction) and subclinical myocardial injury,^35^ and identifies patients at high risk of MACE, including cardiac death.^36,37^

#### Cardiac magnetic resonance

Cardiac magnetic resonance (CMR) can be used to quantify myocardial perfusion in a similar way to positron emission computed tomography (PET) but the post-processing technique is technically demanding and time-consuming. As with positron emission computed tomography, the imaging protocol includes rest and load, with load imaging both performed after injection of gadolinium contrast. After post-processing of resting and loading images, regional and overall myocardial perfusion can be quantified using semi-quantitative (myocardial perfusion reserve index) or fully quantitative (CFR) models. A gadolinium-free loading CMR method using T1 mapping has also recently been proposed for the diagnosis of myocardial ischemia with or without obstructive CAD.^38^ CMR has the advantage of high spatial resolution for transmural characterization of myocardial blood flow and the absence of ionizing radiation, assessment of CMD by a reduction in the myocardial perfusion reserve index, a comprehensive assessment of cardiovascular structure and function, and has been shown to be predictive of prognosis.^39^ However, data remain limited.

#### Left anterior descending coronary Doppler echocardiography

LAD coronary Doppler echocardiography can be used to quantify coronary blood flow velocity at rest and during vasodilator loading. Coronary blood flow velocity is measured by pulsed-wave Doppler and assessed as peak diastolic blood flow velocity at rest and maximal filling. Coronary flow velocity reserve is calculated as the ratio of coronary flow velocity at maximum congestion to coronary flow velocity at rest. The advantages of this technique are that it is low cost, free of ionizing radiation, and widely available, but accuracy is susceptible to operator proficiency and requires echocardiographic visualization of the proximal coronary arteries, which can be a significant challenge in obese adults. There is growing evidence that a reduced coronary flow velocity reserve index can help identify and risk stratify CMD.^40,41^

#### Dynamic Myocardial Perfusion Computed Tomography

Dynamic myocardial perfusion CT can be used to estimate myocardial blood flow in a manner similar to CMR perfusion imaging. Dynamic CT scanning is performed after the injection of an iodinated contrast agent and utilizes dynamic image acquisition to derive an estimate of myocardial blood flow in the same way as CMR.^33^ The main advantages of this technique are the high spatial resolution of CT and the ability to provide accurate anatomical and functional assessment of the myocardium and coronary arteries in a single examination. However, these advantages come at the cost of a higher radiation dose to the patient.

### Treatment

#### Statins

In small trials, these treatments improved angina symptoms, myocardial perfusion, coronary endothelial function, and microvascular function. Statins reduce lipid-rich cores in plaques, inflammation, macrophage, and foam cell formation, promote fibrous cap thickening, and reduce platelet reactivity.^42-44^ Statins are effective in lowering LDL levels, thereby reducing cardiovascular risk. In addition, statins may have multiple effects, including reducing vascular inflammation and improving endothelial function. Follow as Current CMD treatments focus on controlling risk factors and relieving symptoms, and there is a lack of high-level evidence-based recommendations. Table 2 summarises several treatment for CMD.

**Table 2:** Treatment of Coronary Microcirculation dysfunction

Treatment of Coronary Microcirculation Dysfunction	
Drugs	Mechanism
Statins	reducing vascular inflammation and improving endothelial function.
ACEIs	indirectly mediate the effects on coronary microvascular function by lowering blood pressure
Calcium channel antagonists	improve coronary microcirculatory vasodilatation capacity and reduce cardiac afterload
The phosphodiesterase inhibitor	dilated coronary arteries and improved endothelium-dependent vasodilation
β blockers	regional CFVR increased significantly in stenotic segments
Ivabradine	improve exercise tolerance, prolong the ischemic time during exercise

4 ACEIs: angiotensin-converting enzyme inhibitors; CFVR: coronary flow velocity reserve.

In a single-blind, randomized, placebo-controlled study by Kayikcioglu et al.,^45^ 40 patients with microvascular angina were randomly assigned to take either pravastatin (40 mg/day) or placebo. After 3 months of treatment, brachial artery flow-mediated dilation was significantly improved in the pravastatin group. Similarly, exercise duration and 1-mm-ST inhibition were significantly prolonged after statin treatment compared with placebo. In a similar study, Fabian et al. evaluated 40 patients with microvascular angina who had mild hypercholesterolemia and were randomly assigned to placebo (20) or simvastatin 20 mg/day (20).^46^ At the end of the study, brachial artery flow-mediated dilatation was significantly increased in the treatment group, and the time to >1-mm-ST-segment depression on stress testing was significantly prolonged. More recently, Zhang et al. evaluated the effect of combination therapy with statins and calcium channel blockers versus monotherapy in patients with microvascular angina.^47^ 68 Patients were randomized into three groups: fluvastatin (40 mg/day, 23), diltiazem (90 mg/day, 22), and combination fluvastatin (40 mg/day) and diltiazem (90 mg/day, 23). Coronary flow reserve improved in all three groups after 90 days. In addition, the time to ST-segment depression of 1 mm was significantly longer in all groups. Improvement in coronary flow reserve and prolongation of the time to 1-mm ST-segment depression were more pronounced in the combination group than in patients receiving monotherapy. Based on these studies, we recommend the use of statins in most patients with Microvascular disease, unless serious side effects or contraindications exist.

#### Angiotensin-converting enzyme inhibitors

In a study of active medications for RAAS (including ACE inhibitors) with patients with CMD using Doppler measurements of RFC in intracoronary arteries, a randomized controlled trial of ACEI therapy for CMD confirmed the benefit of enalapril. In diabetic subjects, ACEI improved CFR, and in hypertensive patients, ACEI was shown to improve coronary microvascular function by PET,^48^ but no effect was found in the study by Kawata et al.^49^. Non-randomized studies using different methods to assess coronary microvascular function in small samples of patients have further confirmed that ACEIs improve coronary microvascular function; whether ACEIs indirectly mediate the effects on coronary microvascular function by lowering blood pressure is uncertain, but this may not be a single mechanism of benefit, given the key role of angiotensin II in vascular function.^50^ Other evidence suggests that ACEIs are associated with improvements in markers of endothelial function and several circulating biomarkers. In another randomized, double-blind, placebo-controlled trial in female patients with ischemia with non-obstructive coronary artery disease (INOCA), the addition of a selective aldosterone receptor antagonist (eplerenone) to an ACEI did not reduce angina symptoms or improve CFR.^51^

#### Calcium channel antagonists

Calcium channel antagonists are widely used in the treatment of vasospastic angina (VSA). Benidipine, a long-acting dihydropyridine, has produced beneficial prognostic effects in patients with VSA.^52^ Calcium antagonists improve coronary microcirculatory vasodilatation capacity and reduce cardiac afterload and are therefore frequently used in patients with CMD. In addition, long-acting nifedipine may exert cardioprotective effects by inhibiting vascular inflammation and improving endothelial function in patients with Angina or ischemia with no obstructive coronary disease (ANOCA/INOCA).^53^ However, the use of amlodipine in INOCA patients did not significantly improve angina pectoris, and in another study, verapamil failed to reduce ST-segment changes on the ECG due to ischaemia.^54^ Thus, long-acting L-type calcium channel blockers appear to be more favorable to coronary microcirculation than short-acting L-type calcium channel blockers.

#### The phosphodiesterase inhibitor

The phosphodiesterase (PDE) type 3 inhibitor, cilostazol, is used in intermittent claudication and for the prevention of post-stroke, coronary stent restenosis, and percutaneous coronary intervention. The PDE-3 receptor is subdivided into PDE-3A, which is found in platelets, vascular smooth muscle cells, and cardiomyocytes, and PDE-3B, which is found in adipocytes, hepatocytes, pancreatic β-cells, and macrophages. The latter is found in adipocytes, hepatocytes, pancreatic β-cells and macrophages. Inhibition of PDE increases intracellular cyclic AMP, which has antiplatelet, anti-inflammatory, and vasodilatory effects. Cilostazol reduces superoxide anion and improves local hepatocyte growth factor production as well as epithelial and endothelial cell motility and morphogenesis.^55^ The addition of cilostazol appeared to be effective in a prospective multicentre study of patients with nitrate-refractory VSA induced by calcium antagonists and spontaneous or ergonovine.^56^ In a multicentre randomized, double-blind, placebo-controlled trial of amlodipine in patients with treatment-refractory VSA, cilostazol reduced the frequency and intensity of anginal episodes without serious adverse effects.^57^ In a canine model, a PDE -5 type inhibitor (sildenafil) improved perfusion of hypoperfused myocardium during exercise.^58^ In patients with CAD, a single dose of sildenafil (no placebo comparison) dilated coronary arteries and improved endothelium-dependent vasodilation.^59^

#### β blockers

Although early studies using propranolol and vinblastine failed to report beneficial effects,^60,61^ studies using atenolol have reported favorable results in terms of symptoms and exercise stress test parameters.^62^ Atenolol is often prescribed in doses up to 100 mg per day. More recently, reports evaluating the effects of nebivolol in such patients have similarly shown benefit at doses of up to 5 mg per day.^63^ Beta-blockers are particularly effective in patients with high resting heart rates or increased sympathetic tone^64^ and should be avoided in patients with comorbid vasospastic disorders.^65^ Thus, depending on the clinical presentation and comorbidities, beta-blockers can often be an effective treatment of CMD and are used as first-line agents, depending on the clinical presentation and co-morbidities.

One study found significant improvement in coronary microvascular function after treatment with carvedilol compared with treatment with metoprolol in patients with hypertension.^66^ Koepfli et al.^66^ found no effect of carvedilol or metoprolol in stenotic and remote segments of coronary arteries in patients with CAD and stable angina pectoris, but a pooled analysis showed that regional CFVR increased significantly in stenotic segments.^67^ However, whether this effect was due to regression of atherosclerosis or improved coronary microvascular function remains unknown.

#### Ivabradine

Ivabradine does not cause vasoconstriction or negative inotropic effects compared to β-blockers. The beneficial effects of ivabradine on ischaemic heart disease (IHD) are mediated through indirect effects, which improve exercise tolerance, prolong the ischemic time during exercise and reduce the severity and improve exercise tolerance, and prolong the ischaemic time during exercise frequency of angina in patients with stable angina. Ivabradine improved symptoms in patients with primary microvascular angina pectoris, but coronary microvascular function was not altered, suggesting that the improvement in symptoms may be attributable to a heart rate-lowering effect. Other studies have found that ivabradine stabilizes CFR in patients with CAD.^68^ Therefore, channel blockers may have a role in patients with CMD, but further studies are needed.

## Conclusions

CMD is a class of diseases caused by structural and/or functional coronary microvascular disturbances with specific pathogenesis and diagnostic procedures. Assessment of coronary microcirculatory function should be the focus of targeted therapy in patients in whom severe stenosis or spasm of the epicardial coronary arteries has been excluded but who continue to experience episodes of angina. At present, treatment of CMD is mainly risk factor control or treatment of the primary disease, and limited pharmacotherapy remains to be confirmed by further clinical trials.
